# Efficacy of Liposuction in the Treatment of Lipedema: A Meta-Analysis

**DOI:** 10.7759/cureus.55260

**Published:** 2024-02-29

**Authors:** Alexandre C Amato, Juliana L Amato, Daniel Benitti

**Affiliations:** 1 Department of Vascular Surgery, Amato - Instituto de Medicina Avançada, Sao Paulo, BRA; 2 Department of Gynecology, Amato - Instituto de Medicina Avançada, São Paulo, BRA; 3 Department of Vascular and Endovascular Surgery, Medical Valens Center, São Paulo, BRA

**Keywords:** large-volume liposuction, liposuction, meta-analysis, liposuction complication, lipedema

## Abstract

Lipedema, a chronic and painful disorder primarily affecting women without a definitive cure, has traditionally been managed with conservative therapy, notably complete decongestive therapy, across many countries. Recently, liposuction has been explored as a potential surgical treatment, prompting this study to evaluate its effectiveness as possibly the first-line therapy for lipedema. Through extensive literature searches in databases such as CrossRef, Web of Science, PubMed, and Google Scholar up to December 2023, and using the Newcastle-Ottawa Scale for quality assessment, the study selected seven studies for inclusion. Results showed significant post-operative improvements in spontaneous pain, edema, bruising, mobility, and quality of life among lipedema patients undergoing liposuction. However, over half of the patients still required conservative therapy after surgery. Despite these promising results, the study suggests caution due to lipedema's complexity, significant reliance on self-reported data, and limitations of the studies reviewed. Thus, while liposuction may offer symptomatic relief, it should be considered an adjunct, experimental therapy rather than a definitive cure, emphasizing the need for a comprehensive approach to care.

## Introduction and background

Lipedema is a chronic condition primarily marked by the excessive accumulation of subcutaneous fat in the upper and lower legs due to hyperplasia and hypertrophy, as documented in studies [1-3]. This condition predominantly affects women, with a smaller number of cases reported in men [4]. A significant familial predisposition has been noted, highlighting a potential genetic component in its occurrence [4]. Despite its prevalence, lipedema is not yet listed in the World Health Organization's International Classification of Diseases (ICD-10), complicating efforts to accurately determine its global frequency. Current research indicates that it may impact around 12.3% of the adult female population [3,5], with noted variations in prevalence across different regions [4,6].

Lipedema can be classified according to two distinct criteria: fat distribution and morphological appearance. Regarding fat distribution, lipedema is categorized into five types. Type I involves fatty tissue accumulation around the buttocks and hips. Type II is characterized by fat accumulation between the hip and ankle. Type III presents a similar distribution, extending from the hips to the ankles. Type IV is distinguished by the involvement of the arms. Finally, Type V is identified by predominant fatty accumulation in the calf region [7-11].

Morphologically, lipedema is divided into four stages. Stage I is marked by smooth skin with a thickened and softened subcutis, featuring small nodules. In Stage II, larger nodules appear, adding to the thickening and softening of the subcutis. Stage III is characterized by a thick, stiff subcutis with large nodules, accompanied by overhanging masses of tissue on the inner thighs and knees. The most advanced stage, Stage IV, may lead to secondary lymphedema (lipolymphedema), occurring when the fatty deposits impair the lymphatic system [12].

Despite growing research and awareness, the differential diagnosis of lipedema remains a challenge, with the primary diagnosis often based on clinical examination [13]. Consequently, lipedema is frequently misdiagnosed as other conditions like lymphedema or obesity [14], leading to delayed appropriate treatment and progression of the disease, thereby exacerbating the severity of symptoms [15]. 

The current clinical diagnosis of lipedema relies on criteria established by Wold et al. [16] These criteria include: 1) Occurrence exclusively in women; 2) Persistent swelling of the lower limbs, unresponsive to conventional control measures; 3) Bilateral symmetry in the lower limbs with minimal involvement of the feet; 4) Tenderness and pain in the lower limbs upon palpation; and 5) Minimal pitting edema.

In addition to these clinical criteria, pre-operative diagnostic imaging is also employed for staging and classifying the disease. For instance, Amato and colleagues demonstrated the efficacy of ultrasound in measuring the thickness of the cutis and subcutis [11]. Moreover, a systematic review has indicated the utility of other diagnostic tools, such as lymphoscintigraphy, computed tomography scans, magnetic resonance imaging, and magnetic resonance lymphangiography [17].

Although there is currently no definitive cure for lipedema, treatments that provide symptomatic relief and prevent disease progression are available [17,18]. These treatments are categorized into conservative therapy and surgical management [19]. Conservative therapy includes intermittent pneumatic compression and complex decongestive therapy. On the other hand, the surgical management of lipedema primarily involves surgery [20] and liposuction [21]. This approach is considered for individuals with a lipedema diagnosis across all age groups. However, surgery is only considered when conservative therapy fails to yield satisfactory results despite the patient's adherence to the treatment regimen [10].

Liposuction, a key surgical technique in managing lipedema, employs negative pressure to remove fluids and loose fatty tissue through a cannula into a receptacle [22]. Originating from the work of Giorgio Fischer and Aprad in 1976, the procedure was globally popularized by Illouz [23], who developed the wet technique. This method, focusing on removing no more than 1.5L of aspirate from localized fat areas, aimed to minimize blood loss [23]. The field evolved with Klein's introduction of the tumescent fluid infiltration technique (FIT) and Fodor's development of the super-wet technique [24]. More recently, large-volume liposuction (LVL) has emerged, informed by a deeper understanding of liposuction's physiological effects and clinical experiences. Despite its potential for higher complication risks, LVL has seen advancements, including the incorporation of ultrasound and laser guidance to enhance efficacy [25].

Although liposuction can be performed as a straightforward outpatient procedure under local anesthesia, it has not been established as the primary treatment option for lipedema. Given this context, our current meta-analysis was conducted with the aim of evaluating the efficacy of liposuction in treating lipedema. Specifically, we sought to ascertain whether liposuction should be recommended as the first-line therapy for this condition.

## Review

Information sources and searches

A comprehensive search was conducted in CrossRef, Web of Science, PubMed, and Google Scholar databases for records published from 1940 to December 2023. The search strategy involved the use of key terms in various combinations: (liposuction OR surgery OR surgical management OR lipo OR surgical therapy) AND (lipedema OR lipoedema OR adiposis dolorosa OR painful fat). These terms were searched in titles, abstracts, and keywords, with filters applied for language and publication type as needed. Additionally, the bibliographies of relevant studies were manually reviewed to identify further potential sources. However, grey literature was excluded due to its unpublished nature, which could compromise the statistical robustness and scientific integrity of this systematic review and meta-analysis. This decision acknowledges the possibility of missing non-peer-reviewed yet pertinent data.

Eligibility criteria

Two independent reviewers systematically assessed the articles retrieved using pre-established eligibility criteria. Studies were included if they were published in English, specifically evaluated the efficacy of any type of liposuction in treating lipedema, and reported clinical outcomes such as pain, bruising, edema, quality of life (QoL), complications, and the necessity for post-operative conservative therapy. Exclusion criteria encompassed studies that did not meet these parameters or were case series, systematic reviews, conference abstracts, letters to the editor, animal/experimental studies, guidelines, or case reports. Any disagreement between the reviewers was initially resolved through constructive discussions, with the involvement of a third reviewer, if necessary, to reach a consensus.

Data extraction and evaluated outcomes

Two impartial reviewers systematically assessed the selected articles and extracted necessary data for review and analysis. This included author ID (surname of the primary author and publication year), study setting (country), study design, pertinent characteristics of enrolled patients (such as age, sample size, average volume of fluid suctioned, body mass index (BMI), average weight), follow-up period, and reported outcomes. A standardized form was employed for this extraction, and any discrepancies were initially addressed through discussion between the reviewers, with a third reviewer consulted in unresolved cases.

The primary outcomes of interest in this review were post-operative pain, bruising, QoL, edema/swelling, and mobility impairment. These outcomes were assessed based on the measurement tools or scales used in the studies. Additionally, the necessity for post-operative conservative therapy and the safety of liposuction therapy, including the rate and nature of any complications or adverse events, were evaluated. Our analysis extended to explore secondary dimensions of interest, specifically the impact of liposuction on inflammatory markers, the progression of inflammatory diseases, and inflammatory symptoms.

This study was conducted in strict accordance with the Preferred Reporting Items for Systematic Reviews and Meta-Analyses (PRISMA) guidelines. Adherence to these guidelines was intended to ensure methodological integrity, transparency, and replicability of the systematic review and meta-analysis. The PRISMA protocol guided the identification, selection, data extraction, and quality assessment of included studies, as well as data synthesis and results presentation. This methodological approach emphasizes the importance of systematization and standardization in the review process to provide reliable and accurate evidence on the impact of liposuction in treating lipedema.

Quality appraisal

The methodological quality of studies included in this systematic review was rigorously assessed using the Newcastle-Ottawa Scale (NOS). This scale allows for a comprehensive evaluation of studies based on three domains: selection of study groups, comparability of groups, and assessment of outcomes. Each study was independently reviewed, and a maximum of one star was awarded for each criterion fully met within these domains, with the total number of stars reflecting the overall quality.

To further categorize the methodological robustness of these studies, the NOS scores were converted into the Agency for Healthcare Research and Quality (AHRQ) standards, classifying studies as 'poor,' 'fair,' or 'good.' This conversion was based on a predefined scale correlating NOS scores with AHRQ quality levels.

Inter-rater reliability was ensured by resolving any discrepancies in quality assessment through discussion between reviewers or consultation with a third reviewer if necessary.

Data synthesis

In this study, we utilized Review Manager (RevMan 5.4.1, Cochrane, London) and Comprehensive Meta-Analysis software to perform the statistical analyses. We analyzed outcomes such as pain, bruising, edema, QoL, and mobility impairment, which were measured on a continuous scale. To evaluate the overall effect of the intervention, we calculated the mean difference (MD) between pre-intervention and post-intervention values. For the rate of patients requiring post-operative conservative therapy, we expressed this as a percentage of the total patient cohort analyzed. Given the expected high level of variability among study results, we adopted the DerSimonian and Laird random effects model, which provides more conservative estimates of effect sizes in the presence of substantial heterogeneity [26]. To quantify the variability in effect sizes across studies, we calculated the I2 statistic; values exceeding 50% were considered to indicate significant heterogeneity. Statistical significance was established at a 95% confidence level, with a p-value threshold of less than 0.05 (p<0.05) for determining significance. Additionally, we conducted subgroup analyses based on the type of assessment tool used, such as the Visual Analog Scale (VAS), Numeric Rating Scale (NRS), and Likert scale, whenever the data allowed.

Study selection

**Figure 1:**
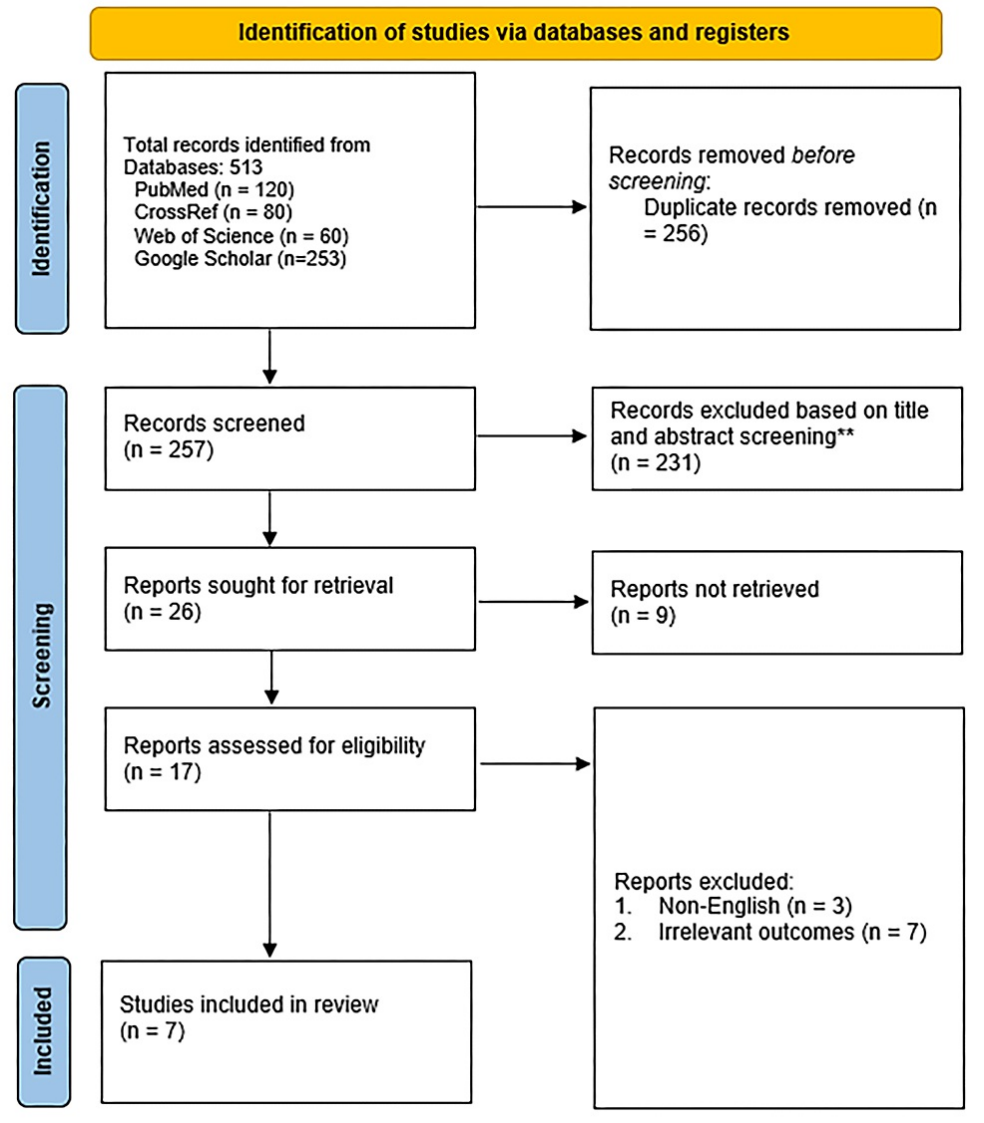
PRISMA flow diagram for study selection. PRISMA: Preferred Reporting Items for Systematic Reviews and Meta-Analyses

Our preliminary search through databases and manual efforts yielded 513 potential articles. An in-depth screening for duplicates led to the exclusion of 256 articles. Subsequent screening based on titles and abstracts resulted in the exclusion of 231 articles. Additionally, nine articles were not retrieved for further evaluation because they were not available in full text and were guidelines, case reports, animal studies, or reviews, leaving 17 articles for potential inclusion. After a detailed assessment, only seven of these articles met our inclusion criteria. The reasons for excluding the remaining 10 articles were as follows: 3 were published in languages not covered by our analysis, and 7 did not report outcomes relevant to our study objectives. The complete selection process and criteria are depicted in Figure 1.

Summary of study characteristics

In our analysis, we included seven studies that collectively involved 451 female patients diagnosed with lipedema. Six of these studies were conducted in Germany, while one study was carried out in Austria. Regarding the assessment tools used to measure outcomes, four studies employed the VAS with a range from 0 to 10, two studies utilized a Likert scale ranging from 0 to 4, and one study used the NRS with a scale from 1 to 10. The duration of follow-up for patients after undergoing liposuction varied significantly across the studies, with a minimum follow-up period of three months and a maximum of 12 years, as detailed in Table 1.

**Table 1:** Summary of study characteristics. NR: Not reported; CDT: Combined decongestive therapy; VAS: Visual analog scale; NRS: Numeric rating scale; BMI: Body mass index

Author ID	Country	Study Design	Patient characteristics					Follow-up period	Assessment tool.	Reported Outcomes
			Sample size	Mean/median age (years)	BMI	Mean Weight (kg)	Aspirate volume (mL)			
Witte et al. 2020 [27]	Germany	Prospective study	63	35	28.4 ± 5.4	NR	12,922 ± 2922	21.5 months	VAS (0 – 10)	Pain, sensitivity to touch, bruising, the feeling of tension, the feeling of heavy legs, sweating, itching, running impairment, occupational impairment, general impairment, and aesthetic impairment. Need for conservative therapy
Schmeller et al. 2012 [28]	Germany	Longitudinal study	112	38.8	NR	79.3	7707 (2564 – 13450)	3 years and 8 months	Likert scale (0 – 4)	Spontaneous pain, pain because of pressure, bruising, restriction of movement, cosmetic impairment, reduction in quality of life, and general impairment. Need for conservative management.
Kirstein et al. 2023 [29]	Germany	Retrospective study	56	40.72	32.61 ± 6.69	93.69 ± 19.9	3831 ± 1971.08	3 months	NRS (1 – 10)	Pain, sensitivity to pressure, limitation to walking, reduction in quality of life.
Dadras et al. 2017 [30]	Austria	Longitudinal study	25	45	35.3	NR	9914 (4000 – 19850)	16 and 37 months	VAS (0 – 10)	Spontaneous pain, pressure sensitivity, tension, bruising, cosmetic impairment, impairment to quality of life, CDT score.
Baumgartner et al. 2021 [31]	Germany	Longitudinal study	60	54.1	NR	79.7	NR	4,8, and 12 years	Likert scale (0 – 4)	Spontaneous pain, sensitivity to pressure, edema, bruising, restriction to impairment, reduction in quality of life, and overall impairment.
Rapprich et al. 2010 [32]	Germany	Prospective study	25	38	NR	NR	2482 ± 968	6 months	VAS (0 – 10)	Pain, bruising, tension, itching, quality of life, edema.
Wollina and Heinig 2019 [33]	Germany	Longitudinal study	111	44	NR	NR	4700 ± 7579	2.0 ± 2.1 years	VAS (0 – 10)	Pain.

Methodological quality assessment

The comprehensive assessment of methodological quality, conducted using the NOS, is detailed in Table 2. Our evaluation revealed that six of the included studies were determined to have 'fair' methodological quality, while one was assessed as having 'poor' methodological quality. A significant limitation identified was that none of the studies achieved more than 2 stars in the 'selection' domain. This was primarily due to their reliance on self-assessment tools, which introduces concerns regarding the reliability of reported outcomes. Additionally, the fact that these studies were carried out in single-center settings limits their generalizability to broader populations worldwide. Furthermore, one study was awarded only one star in the 'outcome' domain, attributed to its relatively short follow-up period of three months. This duration is considered insufficient to conclusively determine the long-term efficacy of liposuction in treating lipedema.

**Table 2:** Methodological quality assessment using the Newcastle-Ottawa scale.

Author ID	Selection (/4)	Comparability (/2)	Outcome (/3)	Methodological Quality
Witte et al. 2020 [27]	2	1	2	Fair
Schmeller et al. 2012 [28]	2	1	2	Fair
Kirstein et al. 2023 [29]	2	1	1	Poor
Dadras et al. 2017 [30]	2	1	2	Fair
Baumgartner et al. 2021 [31]	2	1	2	Fair
Rapprich et al. 2010 [32]	2	1	2	Fair
Wollina and Heinig 2019 [33]	2	1	3	Fair

Spontaneous pain

**Figure 2:**
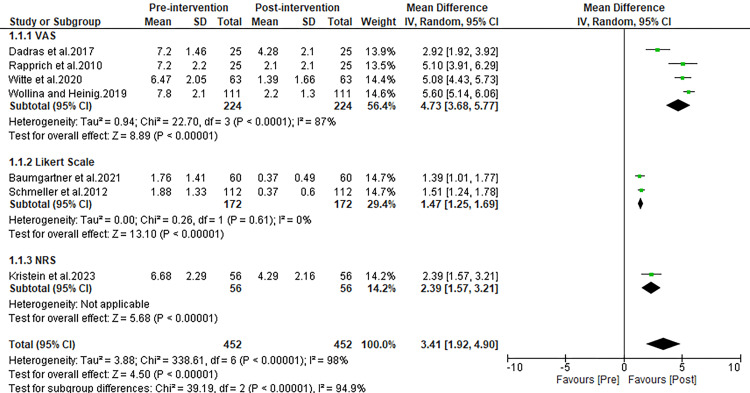
Forest plot comparing pre and post-operative complaint scores for spontaneous pain. Witte et al. 2020 [27], Schmeller et al. 2012 [28], Kirstein et al. 2023 [29], Dadras et al. 2017 [30], Baumgartner et al. 2021 [31], Rapprich et al. 2010 [32], Wollina and Heinig 2019 [33]

Our analysis across seven studies demonstrated a notable decrease in spontaneous pain post-liposuction. The synthesis of these studies highlighted a substantial drop in mean pain scores from pre- to post-surgery (mean D\difference (MD): 3.41; 95% CI: 1.92 to 4.90; p<0.00001). Subgroup analyses further confirmed this finding across various assessment tools, with significant pain score reductions observed with the VAS (MD: 4.73; p<0.00001), the Likert scale (MD: 1.47; p<0.00001), and the NRS (MD: 2.39; p<0.00001). These results, detailed in Figure 2, underscore liposuction's effectiveness in alleviating spontaneous pain in lipedema patients.

Edema/swelling

**Figure 3:**
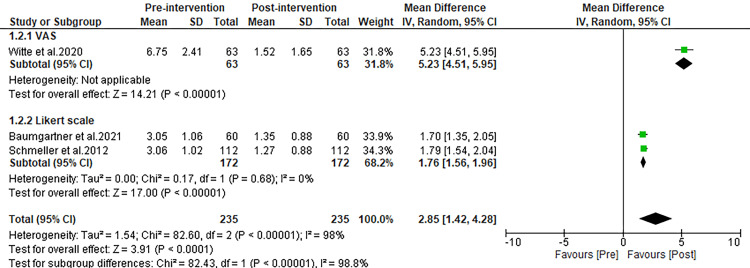
Forest plot comparing pre and post-operative complaint scores for edema/swelling. Witte et al. 2020 [27], Schmeller et al. 2012 [28], Kirstein et al. 2023 [29], Dadras et al. 2017 [30], Baumgartner et al. 2021 [31], Rapprich et al. 2010 [32], Wollina and Heinig 2019 [33]

In this analysis, only three of the included studies have examined the impact of liposuction on edema (swelling). The aggregated data from these studies indicated a significant decrease in edema scores following liposuction treatment, with the pooled MD showing a reduction of 2.85 (95% Confidence Interval (CI): 1.42 to 4.28; p<0.00001), as depicted in Figure 3. Additionally, analysis of mean post-operative edema scores, compared to pre-operative scores, demonstrated significant reductions across different assessment tools. Specifically, there was an MD of 5.23 (p<0.00001) when using the VAS and an MD of 1.76 (p<0.00001) with the Likert scale, highlighting the consistent effectiveness of liposuction in reducing edema, as shown in Figure 3.

Bruising

**Figure 4:**
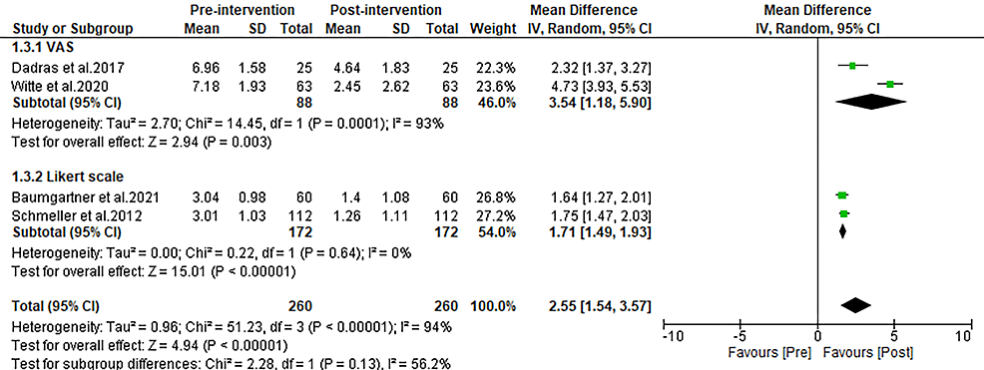
Forest plot comparing pre and post-operative complaint scores for bruising. Witte et al. 2020 [27], Schmeller et al. 2012 [28], Kirstein et al. 2023 [29], Dadras et al. 2017 [30], Baumgartner et al. 2021 [31], Rapprich et al. 2010 [32], Wollina and Heinig 2019 [33]

Among the studies included in our analysis, four reported on the reduction of bruising as assessed by mean scores following liposuction. The combined analysis of these studies revealed that post-operative bruising scores were significantly reduced compared to pre-operative levels, with an MD of 2.95 (95% Confidence Interval (CI): 1.54 to 3.57; p<0.00001), as illustrated in Figure 4. This significant reduction in bruising scores post-liposuction was consistent across the different assessment tools used in the studies. Specifically, the MD was 3.45 (p<0.00001) when measured with the VAS and 1.71 (p<0.00001) with the Likert scale, indicating that liposuction effectively reduces bruising regardless of the measurement method, as shown in Figure 4.

Reduction in mobility impairment

**Figure 5:**
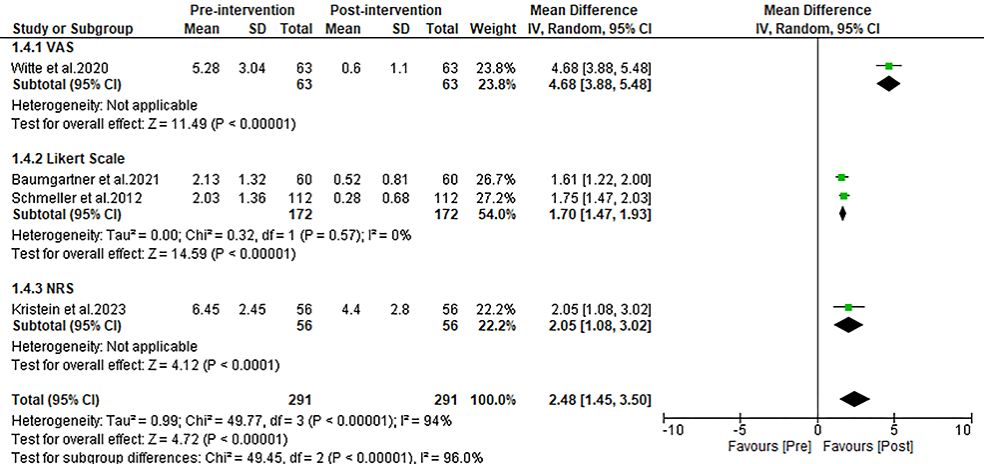
Forest plot comparing pre and post-operative complaint scores for mobility impairment. Witte et al. 2020 [27], Schmeller et al. 2012 [28], Kirstein et al. 2023 [29], Dadras et al. 2017 [30], Baumgartner et al. 2021 [31], Rapprich et al. 2010 [32], Wollina and Heinig 2019 [33]

Given that complaints of restricted mobility are prevalent among patients with lipedema, assessing the impact of liposuction on this particular complaint is critical. The pooled analysis from four studies that addressed this issue revealed a significant improvement in mobility post-liposuction. Specifically, the MD in complaint scores for mobility impairment showed a notable decrease, moving from pre-operative to post-operative assessments (MD: 2.48; 95% Confidence Interval (CI): 1.45 to 3.50; p<0.00001), as demonstrated in Figure 5.

Reduction in QoL impairment

**Figure 6:**
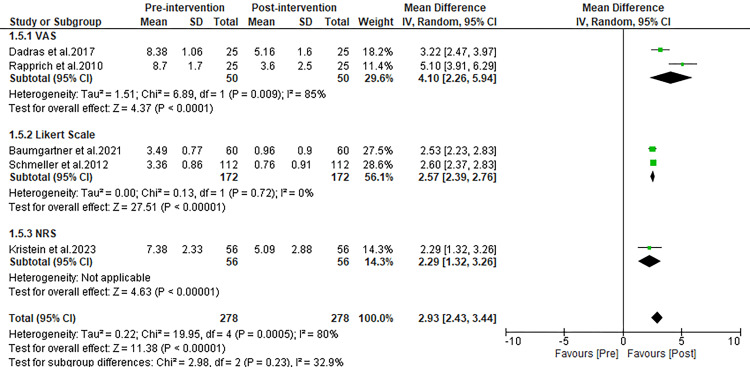
Forest plot comparing pre and post-operative complaint scores for quality of life impairment. Witte et al. 2020 [27], Schmeller et al. 2012 [28], Kirstein et al. 2023 [29], Dadras et al. 2017 [30], Baumgartner et al. 2021 [31], Rapprich et al. 2010 [32], Wollina and Heinig 2019 [33]

Five studies included in our review specifically focused on evaluating the impact of liposuction on the QoL for patients with lipedema. The collective analysis of data from these studies showed a significant improvement in QoL post-liposuction, as evidenced by a notable decrease in the scores measuring QoL impairment. The pooled MD indicated a significant improvement, with post-operative QoL scores being substantially higher than pre-operative scores (MD: 2.93; 95% CI: 2.43 to 3.44; p<0.00001), as detailed in Figure 6.

Need for conservative therapy

**Figure 7:**
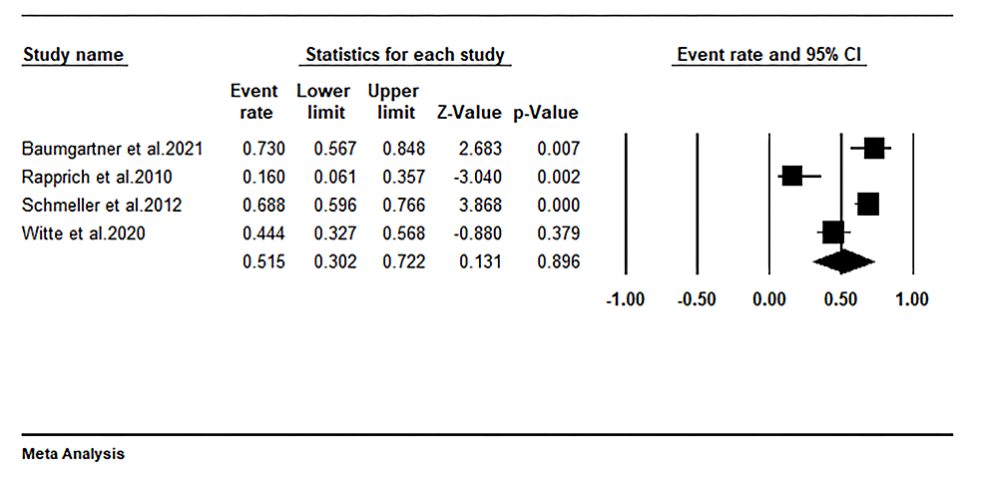
Forest plot showing the proportion of patients using conservative therapy after liposuction. Witte et al. 2020 [27], Schmeller et al. 2012 [28], Baumgartner et al. 2021 [31], Rapprich et al. 2010 [32]

In this study, we found that only four of the included studies reported on the need for conservative therapy following liposuction in lipedema patients. Our pooled analysis of the data from these studies indicates that approximately 51% of patients with lipedema who undergo liposuction continue to require conservative treatments post-surgery, as shown in Figure 7.

Safety

In this study, although four studies reported adverse events following liposuction, pooling of these events was not feasible due to their variability across studies. Wollina and Heinig observed that despite liposuction being well tolerated by lipedema patients, temporary hemoglobinemia was noted in 100% of cases, which was treated with intravenous toluidine blue injections [33]. Furthermore, they reported a temporary burning sensation in 82% of patients; however, this complication resolved without any intervention. Other complications noted in their study included mild arm-vein phlebitis (two patients), an epileptic attack during methemoglobinemia (one patient), microscopic pulmonary fat embolism (one patient), and acute pulmonary edema (one patient). Conversely, Schmeller and colleagues reported that out of 349 liposuctions performed in 112 patients, post-operative wound infections occurred in five cases (1.4% infection rate). Additionally, there was only one case of post-operative bleeding reported on the day of surgery after the removal of 5400ml of fatty tissue from the hips and outer thighs (0.3% bleeding rate) [28]. Rapprich and colleagues reported a single case of deep vein thrombosis, which was promptly treated [32]. Similarly, Dadras and colleagues reported only one case of erysipelas following surgical treatment (complication rate of 1.39%) [30]. Importantly, none of the studies included in our analysis considered the emergence of new symptoms or inflammatory diseases in their post-operative evaluation.

Inflammatory markers

Our investigation sought to delve into secondary dimensions of significant interest, namely the impact of liposuction on inflammatory markers, the progression of inflammatory diseases, and inflammatory symptoms. Despite our efforts to thoroughly analyze the existing literature, we found that the studies included in our review did not fulfill the necessary criteria to provide conclusive answers to these specific questions. The lack of targeted data or sufficiently detailed outcomes related to these secondary dimensions within the studies means that, at present, the evidence base is insufficient to draw definitive conclusions about the effects of liposuction on inflammation-related factors in lipedema patients.

Discussion

In numerous countries, conservative therapy, especially combined decongestive therapy (CDT) is commonly accepted as the initial treatment approach for lipedema. While CDT is effective in halting disease progression and reducing edema, it often fails to prevent the increase in subcutaneous fat and worsening of symptoms in many patients. Consequently, liposuction has emerged as a secondary treatment option, specifically targeting the reduction of subcutaneous fat tissue. The current study demonstrates that liposuction significantly lowers post-operative complaint scores in areas such as spontaneous pain, edema, bruising, mobility impairment, and QoL impairment, compared to pre-operative scores. These results suggest that liposuction can be effective in alleviating symptoms associated with lipedema. However, it is important to note that these findings do not encompass the entire spectrum of lipedema, particularly the inflammatory impact of the condition. This highlights the need for a comprehensive approach to understanding and treating lipedema, considering all its facets.

Improvements in complaints

Pain is a prevalent and debilitating symptom in lipedema, significantly impacting patients' QoL [3]. Our statistical analysis clearly indicates that liposuction leads to a marked reduction in spontaneous pain among lipedema patients. This aligns with the findings of a 2021 retrospective study, which observed a decrease in post-operative pain scores over a 44-hour follow-up period [34]. Additionally, studies such as the one conducted by Dadras et al. support this, demonstrating that liposuction significantly lowers pressure-related pain, with reported pain scores decreasing from 2.91 (indicating strong complaints) pre-operatively to 0.91 (minor complaints) post-operatively [30].

This reduction in pain following liposuction can likely be attributed to two primary factors: the decreased edema in the extremities, resulting from the reduction of subcutaneous space, and the diminution of fat that may serve as a target for inflammation. By reducing the fatty tissue, which is often a site of inflammatory processes in lipedema, liposuction may alleviate the inflammatory component of the disease, thereby reducing pain. Our primary interest in investigating inflammatory markers post-surgery centers on understanding whether the inflammation is reduced or if it merely shifts to target another tissue. This question is fundamental to assessing the efficacy of surgery in addressing the inflammatory component of the condition.

In addition, our meta-analysis has shown a significant improvement in bruising complaints following liposuction, which is in line with previous findings (p<0.00001). This improvement mirrors the results of a prior study conducted in Germany [35]. While the exact mechanism behind the decrease in bruising is not fully understood, it may be related to the amelioration of altered capillary fragility. Reduced capillary fragility leads to a decrease in hematoma formation from minor trauma. Similar observations have been reported in studies examining the effects of decongestive therapy [36].

Our study has shown that liposuction significantly improves mobility in patients with lipedema, as evidenced by the notable decrease in post-operative complaint scores related to mobility impairment compared to pre-operative scores. This suggests that patients experience improved physiological movement after the procedure. A possible reason for this enhancement in mobility is the reduction of skin irritation between the thighs, which can lead to a more balanced and less painful way of walking. The change in gait due to the reduced amount of medial fat in the thighs may also be an important factor in improving mobility. Additionally, the marked reduction in spontaneous pain, pressure-related pain, and edema following liposuction likely plays a significant role in facilitating this improved mobility.

These findings are corroborated by the work of Kirstein and colleagues, who documented significant improvements in the occupational capabilities of lipedema patients after liposuction [29]. Their data indicated that, before liposuction, a substantial portion of patients (43.9%) experienced severe limitations in their work capacity, including 5% who were completely unable to work and 41% who faced moderate occupational disability. Remarkably, after undergoing liposuction, 62% of these patients reported a significant improvement in their ability to engage in physical activities, highlighting the procedure's positive impact on mobility and occupational functionality.

Lipedema often leads to a decline in QoL, particularly in patients experiencing high levels of mental stress [37]. Consequently, it is vital to evaluate whether liposuction can enhance QoL in individuals with lipedema. Our meta-analysis provides evidence that liposuction positively affects overall QoL. This enhancement is largely due to significant improvements in various complaints when considered collectively. In their research, Kirstein and colleagues explored the impact of liposuction on QoL across multiple dimensions [29]. Utilizing the Patient Health Questionnaire-9 (PHQ-9) scale, they discovered that lipedema patients exhibited moderate to severe depression before surgical treatment, which shifted to a mild depressive state post-liposuction. Furthermore, their findings indicate that liposuction also ameliorates physical, psychological, social, and environmental QoL aspects.

Conversely, Dadras and colleagues investigated liposuction's impact on QoL in patients at different stages of lipedema, observing significant QoL improvements post-surgery for both stage II and III patients after mean follow-up periods of 16 and 37 months. However, they noted an increase in QoL impairment between the first and second post-operative follow-ups in stage III patients [30]. This highlights the complexity of liposuction's effects on QoL, suggesting variable outcomes based on the stage of lipedema.

Furthermore, evidence suggests that the benefits of liposuction in alleviating lipedema symptoms can persist for up to 12 years among patients who continue under observation. Baumgartner and colleagues demonstrated that the enhancements observed in spontaneous pain, edema, pressure sensitivity, QoL impairment, bruising, mobility restriction, sensitivity to pressure, and overall impairment were maintained through the 4, 8, and 12-year follow-up periods when compared to pre-operative levels. It was noted, however, that there was a slight increase in impairments from 4 to 8 years post-operatively but not from 8 to 12 years [31]. The stabilization of symptoms from 8 to 12 years was attributed to the fact that patients were significantly older at the 12-year mark than they were at the start of the study.

Need for conservative therapy

We observed that approximately 51% of lipedema patients continue to need conservative therapy post-liposuction, indicating that liposuction does not entirely eliminate the need for such treatments due to ongoing edema. Despite a prevalent reliance on conservative treatments following surgery, data from the studies we reviewed indicate a reduction in the utilization of conservative therapy post-liposuction compared to pre-surgery levels. Schmeller et al. found that of their patient cohort, 67 underwent combined therapy (manual lymphatic drainage (MLD) and compression garments) before liposuction, 18 relied solely on compression garments, and 8 exclusively on MLD [28]. Post-liposuction, 15 patients from the combined therapy group, 5 from the compression garment group, and 4 from the MLD group reported no longer needing any form of conservative therapy. Likewise, Rapprich and colleagues observed that around two-thirds of the patients received MLD and compression therapy before liposuction. Six months following liposuction, only 8% still required MLD, albeit less frequently, and 16% occasionally used compression garments [32]. This suggests that while liposuction doesn't completely negate the need for conservative therapies, it significantly reduces their necessity.

In a 2020 prospective study involving 63 patients with lipedema, it was found that 88.9% underwent manual lymphatic drainage (MLD), and 95.2% used compression garments as part of their pre-surgical management. However, 21.5 months following liposuction, a significantly smaller proportion of patients required MLD (39.7%) and compression garments (31.7%) [27]. Conversely, Dadras et al. [30] assessed the necessity for CDT by calculating a CDT score, which combined the total daily hours of compression garment use with the average number of monthly MLD sessions. Through this CDT score, they identified a significant decrease in the need for conservative therapy among patients in stages II and III of lipedema (p<0.05). Given these findings, we suggest that future research could benefit from employing this CDT scoring method. Such an approach would allow for a more detailed analysis of the ongoing requirement for conservative therapy in lipedema patients, potentially enriching future meta-analyses.

Changes in weight and body shape

While our meta-analysis did not aggregate data on weight changes, liposuction's impact on lipedema patients' weight is an essential consideration. Witte and colleagues observed that liposuction led to a significant reduction in patients' weight by an average of 5.6 kg and a decrease in BMI by 2.3 kg/m^2^ from pre-operative values [27]. This weight loss is attributed to the combination of lifestyle modifications and the permanent removal of fat tissue. However, contrasting results were noted in a long-term study, where 12 years post-liposuction, some patients experienced a weight loss of 6.2 kg, while others (43.3%) saw a weight increase of 7.9 kg above their initial weight [31]. The patients who gained weight were on average nine years older than at the start of the study. This weight gain could be linked to typical physiological changes during menopause, characterized by reduced metabolism, muscle atrophy, and increased fatty tissue accumulation, particularly around the abdomen. Notably, most of these patients had undergone three liposuctions, with an average of 5.5 liters of fat removed per procedure.

Liposuction also leads to changes in the body shape, including reductions in the circumference of hips and extremities. A 2010 retrospective study utilizing 3D imaging showed an average leg volume reduction of 1.2 ± 1.01 liters (6.9%) [32]. Similarly, Schmeller and colleagues reported that, after an average follow-up of three years and eight months, lipedema patients achieved mean reductions of 8 cm in the inguinal region and 4 cm in the calves [28]. Additionally, based on standard clothing sizes, 38% of patients reported a decrease in one size, 25% in two sizes, and 11% in three sizes. These results indicate that successful liposuction can lead to significant morphological improvements and reduce disproportion between the upper and lower body.

Furthermore, it is important to note that none of the studies included in our analysis evaluated changes in visceral fat, which is a known marker of inflammation. The absence of data on visceral fat variation post-liposuction represents a gap in the current literature, as understanding the effect of liposuction on this specific fat compartment could provide valuable insights into the procedure's impact on systemic inflammation and overall metabolic health. This oversight highlights the need for future research to explore the relationship between liposuction, visceral fat reduction, and inflammation markers to more comprehensively assess liposuction's benefits and limitations in treating lipedema.

Caution

Caution is warranted when discussing body fat, a topic often misunderstood by many. Commonly viewed as an unwelcome addition to the body, fat is, for individuals with lipedema, a source of considerable distress rather than fascination. Only a minority of medical professionals recognize adipose tissue as a highly dynamic metabolic organ [38]. However, the last three decades have revolutionized our understanding and perspective of this tissue. Fat is not a singular entity but comprises various anatomical and functional adipose-tissue depots, each with unique characteristics [39].

The challenge of weight regain following weight loss is well-documented [40]. Weight loss triggers significant adaptations within the homeostatic system that regulates body weight, promoting overeating and a return to the original body weight. These adaptations in adipose tissue contribute to a biological inclination to regain weight after loss, including post-liposuction.

Hernandez et al. described fat redistribution following suction lipectomy; one-year post-gluteofemoral liposuction, patients had their fat mass restored but redistributed from the thigh to the abdomen [41]. Similarly, Herbst et al. highlighted the body's defense mechanisms in fat maintenance and restoration post-liposuction, noting tissue growth within and outside the treated areas, with regrowth outside treated areas observed in 61% of women after 6 months [42].

Recent meta-analyses involving over 2.5 million participants by Jayedi et al. have linked central fatness, regardless of overall adiposity, with an increased risk of all-cause mortality. Conversely, larger hip and thigh circumferences are associated with a lower risk [43]. Despite an average obese BMI of 35.3 ± 1.7 kg/m^2^, lipedema is linked to a low risk of diabetes (2%), dyslipidemia (11.7%), and hypertension (13%).

The distribution of body fat, whether in excess or scarcity and whether android/gynoid, significantly affects various reproductive aspects, including fertility and the maintenance of an optimal pregnancy [44-47].

There is a pressing need for patient selection and lifestyle intervention prior to surgical procedures. Without these interventions, most patients will likely experience body fat restoration, preferentially to visceral adipose tissue, which could negatively impact their health and fertility, notwithstanding other improvements.

One of the most significant challenges in studying the surgical outcomes of lipedema is the conflict of interest. Plastic surgeons are motivated to demonstrate the efficacy of their surgical treatments and are likely to publish only positive data that highlight the aspects of the procedure that serve their interests. This bias toward favorable outcomes means that the broader medical and research community may not have access to surgical patients to evaluate their metabolic progression comprehensively. Consequently, articles on lipedema surgery are often utilized as marketing tools to promote more surgical procedures. In many countries, the surgical treatment for lipedema is not included in public health services and is considered a cosmetic, privately funded procedure, introducing a substantial financial bias. The metabolic, inflammatory, and immunological impacts of the surgery may take time to manifest, making the correlation with the surgical procedure less apparent in the short term. Over time, however, evidence is likely to emerge through sporadic case reports, slowly piecing together a more complete picture of the long-term effects of lipedema surgery.

Limitations

This systematic review and meta-analysis come with several limitations that warrant caution in the interpretation of its findings. Many of the studies relied on patient self-assessment for data collection, and there was a high rate of patient dropout during follow-up periods. The loss to follow-up raises concerns that patients who discontinued participation might have experienced complications related to the treatment that were not captured in the study results. Moreover, the reliance on self-assessment by patients poses another limitation, as this method may not effectively identify potential complications related to metabolic changes post-surgery. Another concern is that the outcomes in the included studies rely on subjective self-assessment tools, such as the VAS, NRS, and Likert Scale. These tools have not been validated for evaluating lipedema-specific complaints, casting doubt on the reliability of the reported outcomes. Additionally, the studies included were non-randomized, exposing them to systematic biases inherent to such study designs. Our meta-analyses also encountered high interstudy heterogeneity, likely due to differences in sample sizes, liposuction techniques, follow-up durations, and study designs. Despite this, we mitigated the impact of heterogeneity through the application of a random effects model. Our review was further limited to studies published in English, excluding potentially relevant data from studies in other languages. Notably, nearly all studies were conducted in Germany, raising questions about the applicability of these findings in different international contexts. Furthermore, one study had a follow-up period of only three months, potentially too brief to fully evaluate liposuction's efficacy [29]. Lastly, given that lipedema predominantly affects women and no included study investigated male patients, our findings cannot be extended to the male population.

## Conclusions

Despite the encouraging initial results and a relatively low rate of early complications, considerable caution is warranted. While there appears to be an improvement in spontaneous pain, bruising, edema, and mobility impairment, it is important to recognize that lipedema is a condition far more complex than these criteria alone can encapsulate. The limitations present across all studies are significant. With only 451 published surgical cases of lipedema, most of which were assessed through self-reported questionnaires and marred by a high dropout rate, the evidence is insufficient to deem surgical intervention as the gold standard in treating lipedema.

Surgery should not be considered a cure for lipedema but rather approached as an experimental adjunct therapy. There remains a high percentage of women who require combined conservative treatment. This reality underscores the need for a more cautious and nuanced understanding of lipedema and its management, emphasizing the importance of comprehensive care beyond surgical options.
